# Down-regulation of transcription elogation factor A (SII) like 4 (*TCEAL4*) in anaplastic thyroid cancer

**DOI:** 10.1186/1471-2407-6-260

**Published:** 2006-11-01

**Authors:** Junko Akaishi, Masamitsu Onda, Junichi Okamoto, Shizuyo Miyamoto, Mitsuji Nagahama, Kouichi Ito, Akira Yoshida, Kazuo Shimizu

**Affiliations:** 1 Department of Molecular Biology, Institute of Gerontology, Nippon Medical School, Kanagawa, Japan; 2 Department of Second Surgery, Nippon Medical School, Tokyo, Japan; 3 Department of Surgery, Ito Hospital, Tokyo, Japan; 4 Department of Surgery, Kanagawa Prefectual Cancer Center, Kanagawa, Japan

## Abstract

**Background:**

Anaplastic thyroid cancer (ATC) is one of the most aggressive human malignancies and appears to arise mainly from transformation of pre-existing differentiated thyroid cancer (DTC). However, the carcinogenic mechanism of anaplastic transformation remains unclear. Previously, we investigated specific genes related to ATC based on gene expression profiling using cDNA microarray analysis. One of these genes, transcription elongation factor A (SII)-like 4 (*TCEAL4*), encodes a member of the transcription elongation factor A (SII)-like gene family. The detailed function of *TCEAL4 *has not been described nor has any association between this gene and human cancers been reported previously.

**Methods:**

To investigate the role of *TCEAL4 *in ATC carcinogenesis, we examined expression levels of *TCEAL4 *in ACLs as well as in other types of thyroid cancers and normal human tissue.

**Results:**

Expression of *TCEAL4 *was down-regulated in all 11 ACLs as compared to either normal thyroid tissues or papillary and follicular thyroid cancerous tissues. *TCEAL4 *was expressed ubiquitously in all normal human tissues tested.

**Conclusion:**

To our knowledge, this is the first report of altered *TCEAL4 *expression in human cancers. We suggest that loss of *TCEAL4 *expression might be associated with development of ATC from DTC. Further functional studies are required.

## Background

Anaplastic thyroid cancer (ATC) is one of the most lethal tumors of all human malignancies [1-3]. ATC is well known to often arise from transformation of differentiated thyroid cancer (DTC) of the papillary or follicular type [1-5]. However, little is known about the genetic mechanisms of the transformation of DTC to ATC. We have been attempting to identify new diagnostic markers and drug-target molecules in ATC by cDNA microarray technology, and lately have reported specific changes in expression of several genes in ATCs and cell lines derived from anaplastic thyroid cancers (ACL)s [6].

In this paper, we describe a novel member of the group of down-regulated genes that was identified by expression profiling: transcription elongation factor A (SII)-like 4 (*TCEAL4*). NCBI accession number NM_024863, which is located a Xq22.1. The coding sequence is 1077 basepairs and the mRNA consists of 1516 basespairs according to the NCBI "Gene" database (Gene ID 79921). *TCEAL4 *encodes a member of the transcription elongation factor A (SII)-like (*TCEAL*) gene family. Members of this family contain TFA domains and may work as nuclear phosphoproteins that modulate transcription in a promoter context-dependent manner [7-9]. To date, genetic alterations of *TCEAL4 *in human cancers have not been described. In this study, we examined expression levels of *TCEAL4 *in anaplastic thyroid cancers as well as other types of thyroid cancers and normal thyroid tissue. Expression of *TCEAL4 *was down-regulated in anaplastic thyroid cancers as compared to either normal thyroid tissue or differentiated thyroid cancer tissue. Therefore, we attempted to clarify the mechanism of down-regulation of *TCEAL4 *in ATC, leading to predictions about the function of this gene.

## Methods

### Primary thyroid tissue samples

Primary thyroid cancer tissues were obtained from patients undergoing surgery. Normal thyroid- gland tissues were collected from patients who received surgery for papillary thyroid cancer at Ito Hospital, Tokyo. All patients had given informed consent according to guidelines approved by the Institutional Research Board. Dissected samples were frozen immediately after surgery and stored at – 80°C until needed.

### Cell lines

Eleven cell lines derived from human anaplastic thyroid cancers were used for this study: 8305c, 8505c, ARO, FRO, TTA1, TTA2, TTA3, KTA1, KTA2, KTA3, and KTA4. The cell lines 8305c and 8505c were maintained in Dulbecco's Modified Eagle Medium (Invitrogen, Carlsbad, CA, USA) and ARO and FRO were maintained in Minimum Essential Medium (MEM). The other seven cell lines were grown in RPMI 1640. Papillary thyroid cancer cells (NPA) and follicular thyroid cancer cells (WRO) were cultured in RPMI 1640. All media contained 10% fetal bovine serum (FBS) without antibiotics. The cells were cultured in a 37°C incubator under 5 % CO_2_. In addition, to investigate *TCEAL4 *expression in other cancer cell lines, we examined 91 cancer cell lines, including hepatic cell carcinoma (SK-HEP-1, Hep G2, C-HC-4, Hep-KANO CL2, Hep-TABATA, HuH7, HT17, Li-7, PLC/PRF/5, Hep38, WRL68, Chang Liver, C3A) gastric cancers (HuGC-OOHIRA, AZ521, H-111-TC, SH-10-TC, MKN-7, NUGC-4), colon cancers (DLD-1, SW480, HCT-15, WiDr), pancreatic cancers (MIA Paca2, PK8), breast cancers (MDA-MB-453, CRL1500, YMB-1-E, MCF7, and HBL100), ovarian cancers (CAOV-3, SK-OV-3, OVCAR-3, OV-1063, OVK18), cervical cancers (SIHA, HT-3, D98-AH2, Hela TG, Hela, Ca Ski, Me-180, Hela.P3), lung cancers (RERF-LC AI, PC-14, A549, EBC-1, LU65, LU99, LK-2), urinary bladder cancers (5637, T24, EJ-1), renal cell carcinomas (OS-RC-2, RCC10RGB, VMRC-RCW, Caki-1), bile duct cancers (HuH-28, TFK-1), osteosarcomas (MG-63, Saos02, HuO-3N1, U-2OS), neuroblastoma (IMR-32, NH-12, SCCH-26, NB-1), glioblastomas, brain cancers (TE671, U-138MG, U-373MG, U-118MG, KG-1-C, GI-1, U251, SW1088, Daoy, DBTRG-05MG, D283 Med, A172, T98G, u87MG, u251MG, SNB19, uw18, uw228).

### RNA extraction and cDNA preparation

RNA was extracted as described previously [6]. Briefly, thyroid tissues and cell lines that were collected by trypsinization were homogenized with TRIZOL (Invitrogen, Carlsbad, CA, USA) following the manufacturer's instructions. One microgram of extracted RNA was separated by electrophoresis in 3.0 % formaldehyde denaturing gels to assess RNA quality (RNA degradation). Samples with the ratio of 28S/18S over 1.7 were selected for densitometry. In brief, the intensity of each band was evaluated with Alphaimager (AlphaInonotech, San Leandro, CA, USA). RNA was purified using an RNeasy kit (QIAGEN, Valencia, CA, USA) to eliminate DNA contamination. cDNA was reverse-transcribed from 1 μg of total RNA utilizing standard methodology.

### Semi-quantitative-PCR (SQ-PCR)

To adjust the amount of transcribed cDNA, *GAPDH *was selected as an internal control and SQ-PCR was performed as previously described [6]. All primer sequences are shown in Table 1. All primers were designed with Primer 3 after sequence information was obtained from NCBI GenBank. Each SQ-PCR experiment was performed with 1 μl of cDNA as template, 5U of Takara EX Taq (Takara, Otsu, Japan), 1 × PCR buffer (10 mM Tris-HCl, 50 mM KCl, and 1.5 mM MgCl_2_), 10 nM dNTPs, and 10 pmol each of the forward and reverse primers in a 30 μl total reaction mixture. The PCR conditions 94°C for 2 minutes, followed by, 94°C for 30 seconds, 60°C for 30 seconds, and 72°C for 30 seconds for, 30 cycles in a Thermal Cycler PT-200 (MJ Research Inc., Waltham, MA).

For the evaluation of gene expression between thyroid cancers and normal thyroid -gland tissues, a 2.0% agarose gel was used to separate 10 μl of SQ-PCR product, which was visualized by ethidium bromide staining. The band intensity for each sample was measured by AlphaImager 3300 (AlphaInonotech) after background subtraction. A 16-bit imaging score was acquired from each sample. All SQ-PCR experiments were done in duplicate.

### Quantitative RT-PCR (Q-PCR)

Q-PCR experiments were performed with an ABI PRISM 7700 Sequence Detector (Applied Biosystems, Foster City, CA, USA) according to the comparative threshold cycle (Ct) method following manufacturer's protocol (SYBR Premix Ex Taq™, TAKARA BIO INC., Otsu, Japan). Q-PCR was performed in a 20 ul total reaction mixture in 96-well plates containing; 2 ul of cDNA as template, 10 ul of SYBER Green (Takara, Otsu, Japan), 0.4 ul of ROX dye (Takara, Otsu, Japan), and 0.8 ul of 10 pmol each of the forward and reverse primers. Thermal cycler conditions were 95°C for 30 seconds, followed by, 95°C for 10 seconds and 60°C for 30 seconds, repeated for 40 cycles. All quantitative RT-PCR products were visualized on 2% agarose gels to ensure single products. The A difference in expression between normal thyroid- gland tissue and sample X of an ACL is defined as follows:

ΔC_tX _= C_t-TCEAL4X _- C_t-GAPDHX_

(C_t-TCEAL4, GAPDH _are threshold cycles for amplification of *TCEAL4 *and *GAPDH*, respectively)

ΔC_tN-ave _= Sum of ΔC_tN1 _to ΔC_tN5_/5

(average of ΔCt of five normal thyroid tissues)

ΔΔC_tX _= ΔC_tX _- ΔC_tN_ave_

Expression ratio of *TCEAL4 *to normal thyroid tissue (sample X/average of five normal thyroid tissues) = 2^(-ΔΔCt X)^

### Expression of *TCEAL4 *in normal human tissues

To confirm the expression status of *TCEAL4 *in human organs containing thyroid tissue, we performed SQ-PCR with Human MTC panel 1 & 2 (BD Bioscience, Palo Alto, CA, USA), using SQ-PCR conditions described above.

## Results

### Expression of *TCEAL4 *in primary thyroid tissues

**Figure 1:**
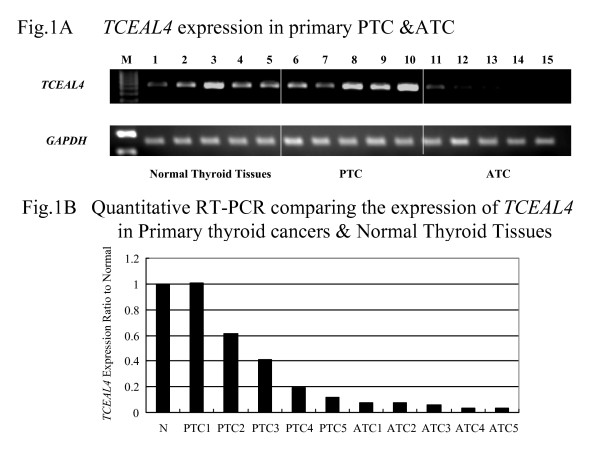
(A) Decreased expression of *TCEAL4 *in ATCs as compared to normal thyroid tissues and PTCs as shown by SQ-PCR. The lane designations are as follows: M, size markers; 1–5, normal thyroid samples, 6–10, primary papillary thyroid cancer samples, 11–15, primary anaplastic thyroid cancer samples. (B) Results of quantitative RT-PCR. The average expression level of *TCEAL4 *among five normal thyroid tissues was set at 1.00, then relative expression ratios were calculated between normal and cancerous tissues.

We examined the expression of *TCEAL4 *in 5 normal thyroid tissues, 5 PTCs, and 5 ATCs by SQ-PCR and Q-PCR. Expression of *TCEAL4 *was significantly down-regulated in ATCs as compared to normal thyroid tissues and PTCs (Figure 1A and 1B). Clinical profiles of patients that had samples subjected to SQ-PCR are shown in Table 2.

### Down-regulation of *TCEAL4 *in ACLs

**Figure 2:**
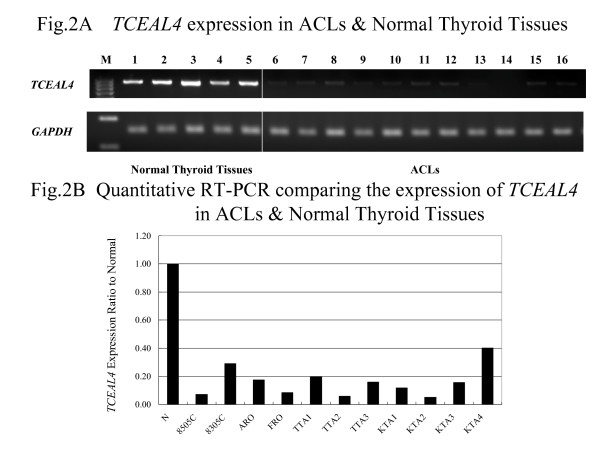
(A) Down-regulation of *TCEAL4 *in ACLs as compared to normal thyroid tissues as shown by SQ-PCR. The following are the lane designations: M, size marker; 1–5, normal thyroid; 6, 8305c; 7, 8505c; 8, ARO; 9, FRO; 10, TTA1; 11, TTA2; 12, TTA3; 13, KTA1; 14, KTA2; 15, KTA3; 16, KTA4. (B) Results of quantitative RT-PCR. *TCEAL4 *expression of normal thyroid is the average of five normal thyroid glands. When the expression of normal thyroid was settled as 1.00, the ratio represented relative expression of *TCEAL4 *in the sample.

We also investigated the expression of *TCEAL4 *in cell lines derived from 11 anaplastic thyroid cancers (ACLs) and five normal thyroid tissues by SQ-PCR and Q-PCR. Expression of *TCEAL4 *was markedly reduced in all ACLs as compared to normal thyroid tissue (Figure 2A and 2B). The expression ratio of *GAPDH *and *TCEAL4 *revealed that *TCEAL4 *was significantly under-expressed in ACLs.

### Expression of *TCEAL4 *in human cancer cell lines

**Figure 3:**
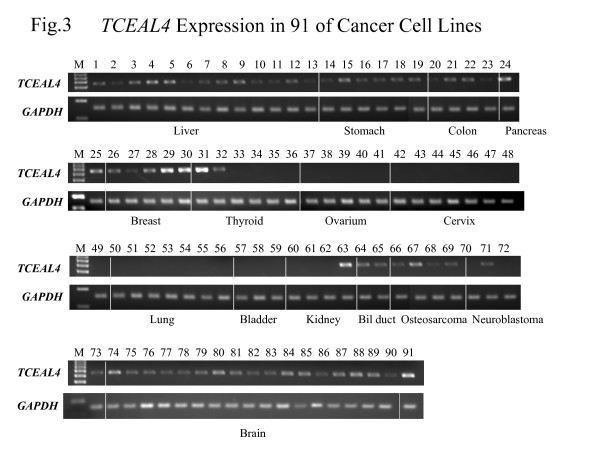
SQ-PCR analysis of the expression of *TCEAL4 *in 91 of cancer cell lines. The lanes are designated as: M, size marker; 1, SK-HEP-1; 2, Hep G2; 3, C-HC-4; 4, Hep-KANO CL2; 5, Hep-TABATA; 6, HuH7; 7, HT17; 8, Li-7; 9, PLC/PRF/5; 10, Hep38; 11, WRL68; 12, Chang Liver; 13, C3A; 14, HuGC-OOHIRA; 15, AZ521; 16, H-111-TC; 17, SH-10-TC; 18, MKN-7; 19, NUGC-4; 20, DLD-1; 21, SW480; 22, HCT-15; 23, WiDr; 24, MIA Paca2; 25, PK8; 26, MDA-MB-453; 27, CDL1500; 28, YMB-1-E; 29, MCF7; 30, HBL100; 31, WRO; 32, NPA; 33, ARO; 34, FRO; 35, 8505c; 36, 8305c; 37, CAOV-3; 38, SK-OV-3; 39, OVCAR-3; 40, OV-1063; 41, OVK18; 42, SIHA; 43, HT-3; 44, D98-AH2; 45, Hela TG; 46, Hela; 47, Ca Ski; 48, Me-180; 49, Hela.P3; 50, RERF-LC AI; 51, PC-14; 52, A549; 53, EBC-1; 54, LU65; 55, LU99; 56, LK-2; 57, 5637; 58, T24; 59, EJ-1; 60, OS-RC-2; 61, RCC10RGB; 62, VMRC-RCW; 63, Caki-1; 64, HuH-28; 65, TFK-1; 66, MG-63; 67, Saos02; 68, HuO-3N1; 69, U-2OS; 70, IMR-32; 71, NH-12; 72, SCCH-26; 73, NB-1; 74, TE671; 75, U-138MG; 76, U-373MG; 77, U-118MG; 78, KG-1-C; 79, GI-1; 80, U251; 81, SW1088; 82, Daoy; 83, DBTRG-05MG; 84, D283 Med; 85, A172; 86, T98G; 87, u87MG; 88, u251MG; 89, SNB19; 90, uw18; 91, uw228.

To assess whether the loss of expression of *TCEAL4 *was specific to ACLs, we examined *TCEAL4 *expression by SQ-PCR in 91 of cancer cell lines including differentiated thyroid cancers derived from papillary and follicular cancers. *TCEAL4 *expression was absent or under-expressed in 46 of 91 (51%) cell lines examined, namely hepatic cell carcinomas, gastric cancers, colon cancers, ACLs, ovarian cancers, cervical cancers, lung cancers, urinary bladder cancers, renal cell carcinomas and neuroblastomas. Interestingly, expression of *TCEAL4 *was markedly reduced in ACLs as compared to DTCs (Figure 3).

### Expression of *TCEAL4 *in human normal tissues

**Figure 4:**
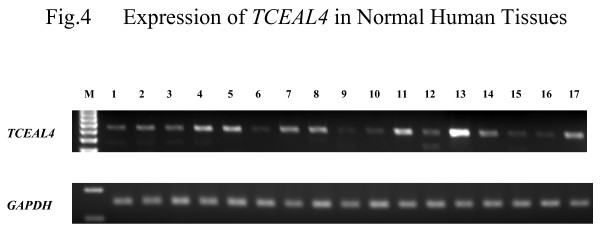
Expression of *TCEAL4 *in normal human tissues. The following are the lane designations: M, size marker; 1, heart; 2, brain; 3, placenta; 4, lung; 5, liver; 6, skeletal muscle; 7, kidney; 8, spleen; 9, pancreas; 10, thymus; 11, prostate; 12, testis; 13, ovary; 14, small intestine; 15, colon; 16, leukocytes; 17, thyroid.

We also examined expression of *TCEAL4 *by SQ-PCR in normal human tissues including normal thyroid gland. *TCEAL4 *was expressed ubiquitously in all normal human tissues tested: heart, brain, placenta, lung, liver, skeletal muscle, kidney, spleen, pancreas, thymus, prostate, testis, ovary, small intestine, colon, leukocytes, and thyroid (Figure 4).

## Discussions and conclusion

ATC has poor prognosis with a one year survival rate whereas PTC has a favorable prognosis [1-3]. It is well known that most ATCs arise from transformation of DTC [1-5], especially PTCs with repeated recurrences in regional lymph nodes [11]. Recently, molecular studies have been developed to analyze ATC. Mutations of TP53 [14,15], beta-catenin [16], and BRAF [17] were detected at a high frequency in ATC. In addition, bcl-2 [18], cyclin D1 [19], Y-box binding protein (YB-1) [20], peroxisome proliferator-activated receptor-γ (PPAR-γ) [21], maspin [22], and haemoglobin beta (HBB) [10] have been reported to play an important role in anaplastic transformation of thyroid cancer. In a previous study, we found frequent allelic losses at 1q, 9p, 11p, 11q, 17p, 19p and 22q in a panel of 21 ATCs suggesting that the region around these chromosome loci might harbor a tumor suppressor gene for ATC [23].

cDNA microarray technology has been developed to analyze a large number of genes in a biological sample at the same time. For the current study, we discovered genetic alterlation related to ATC carcinogenesis [6], and focused on *TCEAL4 *on the basis of cDNA microarray results. The protein encoded by this gene contains one transcription elongation factor A (*TCEAL4*), SII-related family motif. Yeh and Shatkin identified a gene from a Hela cell library encoding p21 that belongs to the SII family [7]. This gene may function as a nuclear phosphoprotein that modulates transcription in a promoter context-dependent manner. The Rous sarcoma virus long terminal repeat (RSV LTR) promoter was decreased by p21 indicating that both the zinc finger-like motif and the arginine/serine-rich region of p21 are essential for inhibition of RSV LTR function [8]. It was reported that p21, or *TCEAL1 *was expressed in all normal human tissues tested by northern blot analysis and this gene is located at Xq22.1 [9]. Multiple family members of *TCEAL4 *are located on chromosome X.

This study showed that mRNA expression of *TCEAL4 *was under-expressed in ATCs and ACLs as compared to normal thyroid tissues and DTCs. Interestingly, *TCEAL4 *was expressed ubiquitously in normal human tissues. Based on these results, we suggest that loss of *TCEAL4 *expression might be associated with development of ATC from DTC or normal thyroid glands. Unfortunately, we could not examine protein expression of *TCEAL4 *in thyroid tissues because an antibody of *TCEAL4 *was not available.

Gene silencing is caused by several reasons, including methylation of promoter region and loss of heterozygosity (LOH). Methylation of DNA promoter region is one of the mechanisms for silencing transcription and inactivating tumor suppressor genes. In this study, we were unable to examine LOH because normal paired DNA samples were unavailable. Instead, we investigated the methylation status of the promoter region of *TCEAL4 *for 11 ACLs as described previously [10] and found that it was mainly hemi-methylated (the data was not shown). We suggest that methylation may be one of the mechanisms for the down-regulation of *TCEAL4 *in ATC and ACL.

In conclusion, this is the first study that describes of altered *TCEAL4 *expression in human cancer. The results suggest that loss of expression of *TCEAL4 *might play a role in anaplastic thyroid cancers. To confirm whether the activity of *TCEAL4 *would suppress cell survival in ATC, further functional analyses are required.

## Competing interests

The author(s) declare that they have no competing interests.

## Authors' contributions

JA carried out the molecular genetic studies and drafted the manuscript. MO participated in the design of the study and helped to draft the manuscript. JO helped to participate in the design of the study. SY helped to participate in the molecular genetic studies. MH and KI provided thyroid materials. AY donated anaplastic thyroid cancer cell lines. KS participated in coordination of the study. All authors read and approved the final manuscript.

## Pre-publication history

The pre-publication history for this paper can be accessed here:



## Figures and Tables

**Table 1:** Primers for semi-quantitative and quantitative RT-PCR

Gene	Forward primer (5'-3')	Reverse primer (5'-3')
*GAPDH*	ggaaggtgaaggtcggagt	tgggtggaatcatattggaa
*TCEAL4 *(semi-quantitative-PCR)	ctggctcattacctcaaggagta	agtggacacagctttcagaattg
*TCEAL4 *(quantitative-PCR)	gaaaaggaggggaaatctcg	ggctttctctcgtcttgtgg

**Table 2:** Clinical profile of patients selected for RT-PCR

**No.**	**Histotype**	**age**	**sex**
1	normal thyroid tissue	25	F
2	normal thyroid tissue	44	F
3	normal thyroid tissue	38	F
4	normal thyroid tissue	23	F
5	normal thyroid tissue	36	F
6	papillary thyroid cancer	27	F
7	papillary thyroid cancer	23	F
8	papillary thyroid cancer	33	F
9	papillary thyroid cancer	53	F
10	papillary thyroid cancer	62	F
11	anaplastic thyroid cancer	64	M
12	anaplastic thyroid cancer	56	M
13	anaplastic thyroid cancer	53	M
14	anaplastic thyroid cancer	71	F
15	anaplastic thyroid cancer	68	M
